# Kidins220/ARMS Is a Novel Modulator of Short-Term Synaptic Plasticity in Hippocampal GABAergic Neurons

**DOI:** 10.1371/journal.pone.0035785

**Published:** 2012-04-26

**Authors:** Joachim Scholz-Starke, Fabrizia Cesca, Giampietro Schiavo, Fabio Benfenati, Pietro Baldelli

**Affiliations:** 1 Department of Neuroscience and Brain Technologies, Istituto Italiano di Tecnologia, Genova, Italy; 2 Molecular Neuropathobiology Laboratory, Cancer Research UK London Research Institute, London, United Kingdom; 3 Department of Experimental Medicine, University of Genova and National Institute of Neuroscience, Genova, Italy; University of Iowa, United States of America

## Abstract

Kidins220 (Kinase D interacting substrate of 220 kDa)/ARMS (Ankyrin Repeat-rich Membrane Spanning) is a scaffold protein highly expressed in the nervous system. Previous work on neurons with altered Kidins220/ARMS expression suggested that this protein plays multiple roles in synaptic function. In this study, we analyzed the effects of Kidins220/ARMS ablation on basal synaptic transmission and on a variety of short-term plasticity paradigms in both excitatory and inhibitory synapses using a recently described Kidins220 full knockout mouse. Hippocampal neuronal cultures prepared from embryonic Kidins220^−/−^ (KO) and wild type (WT) littermates were used for whole-cell patch-clamp recordings of spontaneous and evoked synaptic activity. Whereas glutamatergic AMPA receptor-mediated responses were not significantly affected in KO neurons, specific differences were detected in evoked GABAergic transmission. The recovery from synaptic depression of inhibitory post-synaptic currents in WT cells showed biphasic kinetics, both in response to paired-pulse and long-lasting train stimulation, while in KO cells the respective slow components were strongly reduced. We demonstrate that the slow recovery from synaptic depression in WT cells is caused by a transient reduction of the vesicle release probability, which is absent in KO neurons. These results suggest that Kidins220/ARMS is not essential for basal synaptic transmission and various forms of short-term plasticity, but instead plays a novel role in the mechanisms regulating the recovery of synaptic strength in GABAergic synapses.

## Introduction

Synaptic transmission at fast chemical synapses plays a prominent role in the communication between neurons in the central and peripheral nervous systems. Presynaptic action potentials trigger the fast release of neurotransmitters, which impact on the membrane potential of the postsynaptic cell through activation of specific ligand-gated channels. The efficacy of synaptic transmission for successive action potentials does not remain constant, but it changes depending on the pattern of recent activity. Dynamic alterations lasting from milliseconds to minutes are referred to as “short-term synaptic plasticity” (STP) [1], which is thought to have an important role in the transfer of information between neurons. Synaptic plasticity can manifest itself in several forms, ranging from facilitation to depression, and may vary between cell types or even between synapses of the same neuron. Despite considerable progress in our understanding of the mechanisms underlying STP, many questions remain unanswered, particularly regarding the identity and specificity of the molecular players involved. In addition to their roles in differentiation and survival, neurotrophins (NT) have been recognized as important synaptic modulators [2]. In particular, brain-derived neurotrophic factor (BDNF) has a multitude of functions in the formation, maturation and plasticity of both excitatory and inhibitory synapses [3]. The transmembrane protein Kidins220/ARMS (Kinase D-interacting substrate of 220 kDa/Ankyrin-Rich Membrane Spanning) [4], [5], referred hereafter as Kidins220, has been identified as a direct downstream target of activated neurotrophin receptors. Recent reports have begun to characterize the involvement of Kidins220 in specific neurotrophin effects on synaptic transmission, such as the potentiation of evoked excitatory post-synaptic currents in response to acute BDNF treatment [6] and the enhancement of miniature inhibitory post-synaptic currents upon chronic exposure to BDNF [7]. A similar enhancement of GABAergic input was also observed in Kidins220-overexpressing excitatory neurons, while the opposite effect occurred in cells with reduced Kidins220 expression, leading to the hypothesis that BDNF released from the post-synaptic excitatory neuron may be responsible for the enhancement [7].

**Figure 1:**
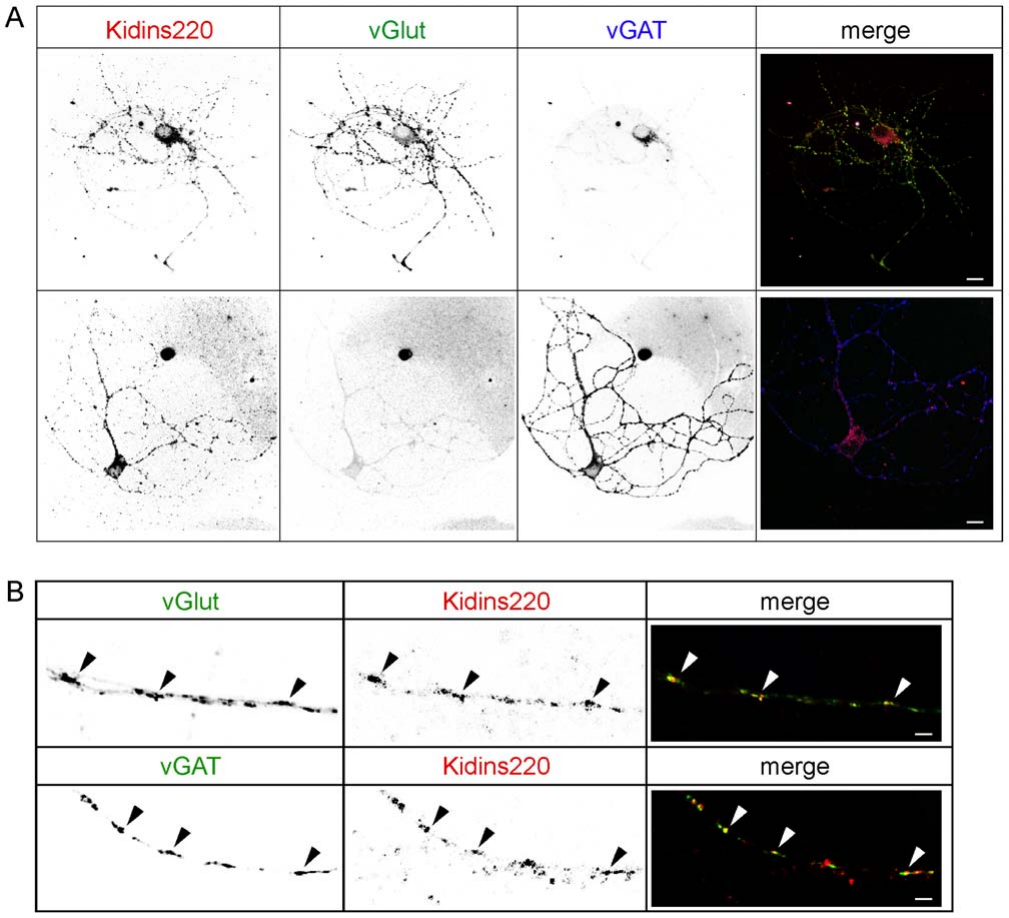
Immunocytochemical analyses showed similar Kidins220 expression and pre-synaptic localization in excitatory and inhibitory autaptic neurons. **A**) Immunofluorescence images of a glutamatergic (vGlut-positive) and a GABAergic (vGAT-positive) autaptic neuron stained with anti-Kidins220 (red), anti-vGlut1 (green) and anti-vGAT (blue) antibodies. Merged images are shown on the right. Scale bars, 10 µm. **B**) Higher magnification of excitatory and inhibitory neuronal processes stained with anti-Kidins220 (red) and anti-vGlut or anti-vGAT (green) antibodies. Merged images are shown on the right. Kidins220 shows a good co-localization with both pre-synaptic markers (arrowheads), indicating pre-synaptic localization of the protein in both excitatory and inhibitory terminals. Scale bars, 1 µm.

Besides its direct interaction with the NT receptors Trks and p75^NTR^ [5], [8], [9], Kidins220 binds to many proteins, such as Rho-GEF Trio [10] and the kinesin-1 motor complex [11]. These findings have lead to the view of Kidins220 as a scaffold protein coordinating diverse regulatory functions at the plasma membrane, via its multiple protein interaction domains. Interestingly, subunits of the NMDA [12] and AMPA receptors [13] are among the identified interacting proteins. This opens the possibility of a NT-independent role of Kidins220 in the modulation of synaptic function. Reduced Kidins220 expression lead to increased excitatory synaptic activity, both in hippocampal cultured cells [14] and acute brain slices [13], and to an increased long-term potentiation of excitatory responses [15]. In addition, Kidins220 regulates the phosphorylation state and cell surface expression of the AMPA receptor subunit GluA1 [13]. These results seem to support a NT-independent role of Kidins220 in the modulation of basal synaptic transmission and plasticity, even though the involvement of NTs was not specifically excluded in the above mentioned studies.

**Table 1:** Analysis of miniature post-synaptic currents.

	mEPSC		mIPSC	
	WT	KO	WT	KO
Number of cells	10	13	12	14
Amplitude (pA)	12.2±0.3	12.3±0.2	28.1±0.8	27.7±0.8
Rise time_10–90_ (ms)	1.4±0.1	1.4±0.1	3.4±0.2	2.9±0.2
Decay time (ms)	5.2±0.4	5.4±0.3	27.6±1.5	24.5±1.3
Frequency (Hz)	0.63±0.12	0.51±0.10	1.28±0.24	1.04±0.21
f range (Hz)	0.24–1.21	0.23–1.43	0.23–2.62	0.27–2.60

Recordings from wild type (WT) and Kidins220*^−/−^* (KO) neurons in the presence of either bicuculline (for mEPSC) or CNQX (for mIPSC) in the bath solution, at a holding potential of −75 mV. None of the analyzed parameters revealed significant differences between WT and KO cells (p>0.05; unpaired Student's t-test).

Furthermore, the amount of Kidins220 protein itself is strongly affected by ongoing synaptic activity, as first demonstrated in rat hippocampal cultures [14]. Subsequent work has shown that Kidins220 is a target of the calcium-dependent protease calpain, activated either by excitotoxic activation of NMDA receptors [12] or chemically induced depolarization [15]. From these results a picture emerges in which the amount of Kidins220 expression and the level of neuronal activity appear to be reciprocally connected.

To date, all studies examining the relationship between Kidins220 and synaptic transmission have relied on acutely modulating Kidins220 levels by either overexpression or downregulation as well as on the use of a Kidins220 knockout strain [16], in which Kidins220^+/−^ mice express 60–70% of the physiological Kidins220 levels. Homozygous knockout animals die at a very early embryonic stage, thus precluding any functional studies [16]. Recently, Cesca *et al.* [6], [17] reported the generation and phenotypic characterization of a new Kidins220-deficient mouse line. In this line, Kidins220^−/−^ embryos survive until late stages of gestation and show distinct areas of cell death and reduced neuronal responsiveness to neurotrophic stimuli. Here, we used embryonic hippocampal cultures from this strain to study the consequences of constitutive Kidins220 ablation on synaptic transmission and short-term plasticity in both excitatory and inhibitory synapses.

**Figure 2:**
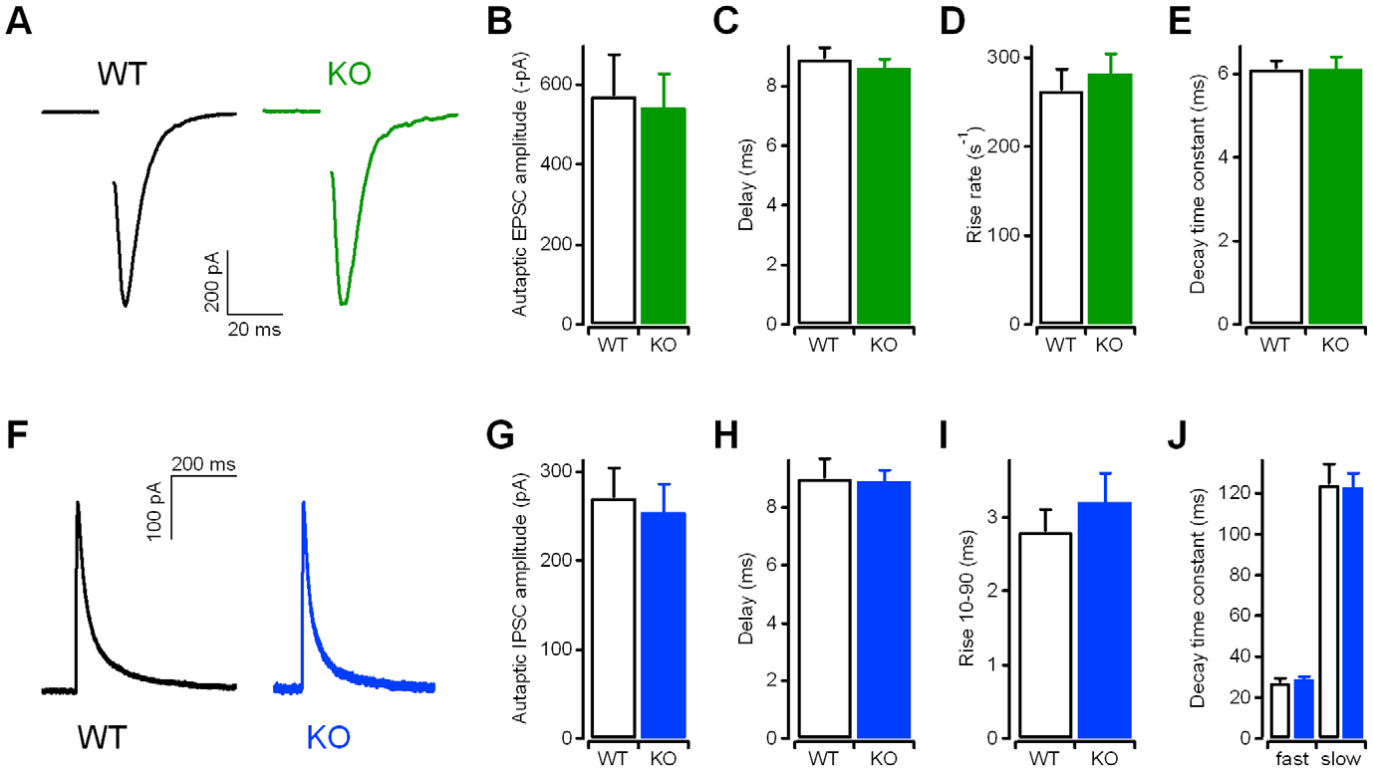
Excitatory and inhibitory post-synaptic currents recorded from autaptic Kidins220^−/−^ neurons showed normal amplitudes and kinetics. **A**) Representative eEPSC recordings in WT and KO autaptic neurons in response to brief depolarization. Stimulus transients have been removed for clarity. Holding potential −86 mV. **B**) eEPSC amplitudes recorded from autaptic neurons (n = 52 for WT; n = 45 for KO). **C**) Delay times were determined as the time between the stimulus and the peak of the EPSC response. **D**) The rise rate as a measure of the activation kinetics was determined from the slope of the EPSC's rising phase. **E**) The time constant of EPSC deactivation was determined by fitting the EPSC decay phase with a mono-exponential function. **F**) Representative eIPSC recordings in WT and KO neurons in response to brief depolarization. Stimulus transients have been removed for clarity. Holding potential −66 mV. **G**) eIPSC amplitudes recorded from autaptic neurons (n = 36 for WT; n = 29 for KO). **H**) Delay times were determined as the time between the stimulus and the peak of the IPSC response. **I**) The rise time (from 10% to 90% of the IPSC amplitude) was determined from the rising phase of IPSC. **J**) Fast and slow time constants of IPSC deactivation were determined by fitting the IPSC decay phase with a bi-exponential function. For the data in **C–E** and **H–J**, n = 20 for both WT and KO. None of the analyses revealed significant differences (p>0.05; unpaired Student's t-test). See the *Methods* section for details on the determination of current kinetics.

## Results

### Excitatory and inhibitory autaptic neurons express Kidins220 to a similar extent

In this study, we used cultured hippocampal neurons for whole-cell patch-clamp recordings of synaptic activity, since the late embryonic lethality of Kidins220^−/−^ mice [6] precluded functional studies on neurons in hippocampal slices of adult animals. Spontaneous neurotransmitter release was evaluated from the input originating from multiple synaptically connected cells in neuronal networks. With the exception of a subset of the data on inhibitory short-term plasticity which derived from extracellular stimulation of GABAergic synapses in neuronal networks, recordings of electrically evoked release were made from autaptic neurons, which offer the advantage to activate a defined homogenous population of monosynaptically connected synapses [18], [20]. In this respect, they are equivalent to paired recordings between two connected neurons, but contrary to these, they allow to record the activity of a neuron's synaptic contacts as a whole, as all contacts generated by axonal sprouting are forced to reach the same post-synaptic target.

**Figure 3:**
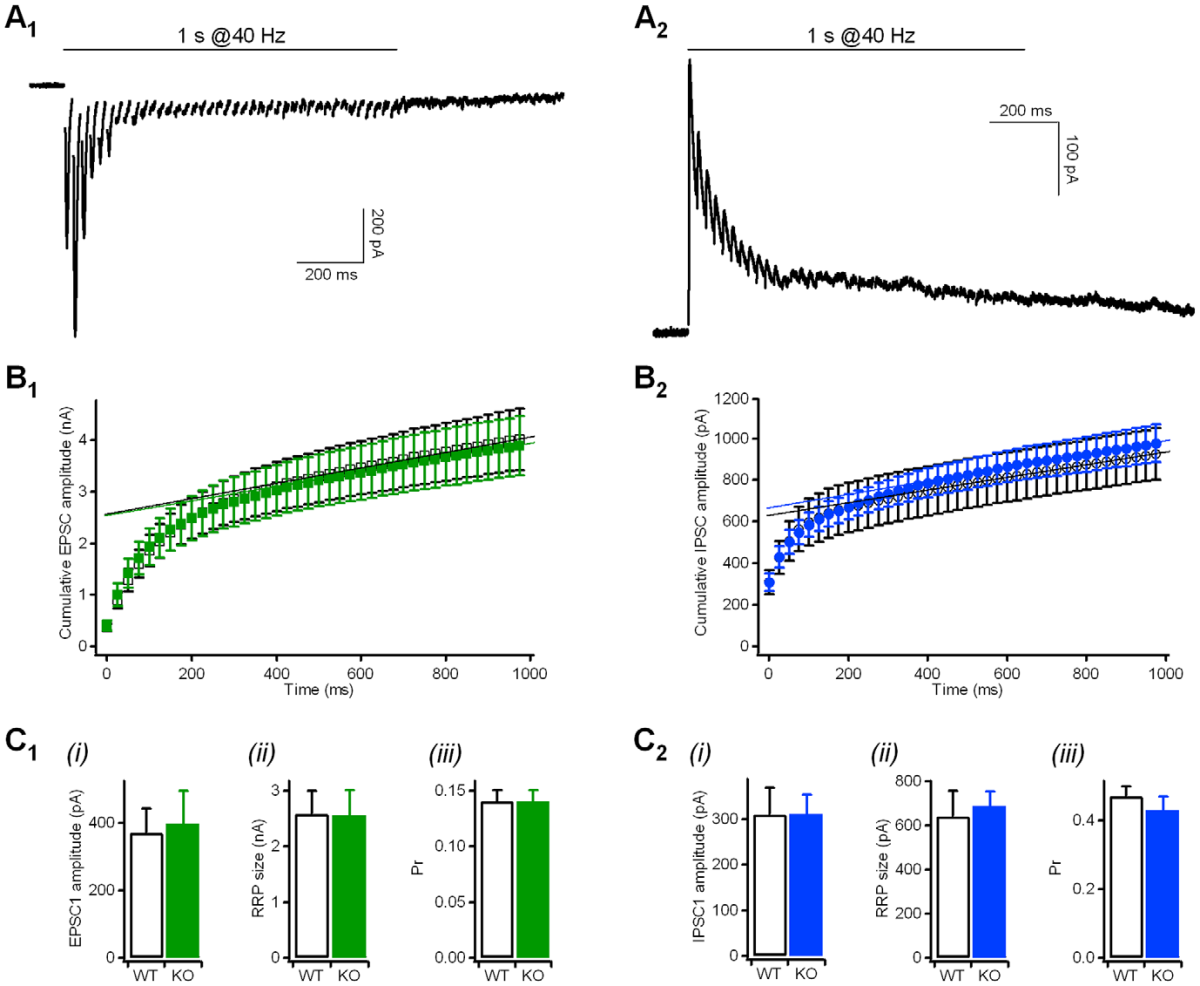
Cumulative amplitude profile analyses did not reveal differences in RRP sizes and vesicle release probabilities between wild type and Kidins220^−/−^ neurons. **A**) Representative current traces (EPSCs in A_1_; IPSCs in A_2_) in response to a 1-s stimulation train at 40 Hz. Holding potential −86 mV in A_1_, −66 mV in A_2_. **B**) Cumulative amplitude profile of EPSCs (B_1_) and IPSCs (B_2_) during repetitive stimulation at 40 Hz for 1 s (see current traces in A). Currents were recorded from autaptic neurons, with n = 16 WT (open squares), n = 15 KO (filled squares) for EPSC data, and n = 13 WT (open circles), n = 16 KO (filled circles) for IPSC data. Data points between 600 and 1,000 ms were subjected to a line fit to estimate the size of the cumulative EPSC/IPSC amplitude before steady-state depression (see below). **C**) The parameters derived from the cumulative amplitude profile analyses in B did not differ between WT and KO neurons (p>0.05; unpaired Student's t-test): (i) the amplitude of the first autaptic EPSC (**C_1_**) and IPSC (**C_2_**) in the train; (ii) the cumulative current amplitude before steady-state depression (indicating the size of the readily releasable pool (RRP)) estimated from the intercept of the line fit (in **B**) at t = 0 s; (iii) the vesicle release probability (Pr) calculated as the ratio between EPSC1/IPSC1 (see i) and the respective RRP size (see ii).

Immunostaining experiments have shown that Kidins220 is expressed in both excitatory and inhibitory neurons of hippocampal cultures [7]. In order to check for Kidins220 expression in autaptic neurons, we performed triple labeling with anti-Kidins220, anti-vGlut and anti-vGAT antibodies (Figure 1A). By this approach, we were able to separately evaluate Kidins220 expression in excitatory (vGlut-positive) and inhibitory (vGAT-positive) neurons. Kidins220 immunoreactivity was present in a punctate staining pattern in cell bodies and processes of both neuronal populations. Close examination of neuronal processes at higher resolution revealed a noticeable level of co-localization of Kidins220 with the excitatory and inhibitory pre-synaptic markers vGlut and vGAT (Figure 1B, arrowheads), thus indicating that the protein is present in the pre-synaptic compartment of both cell types. Quantification of fluorescence intensity showed no differences in the levels of Kidins220 expression between the two groups (data not shown). This analysis confirmed that Kidins220 was expressed ubiquitously and in comparable amounts in excitatory and inhibitory autaptic neurons.

### Kidins220^−/−^ neurons show normal spontaneous neurotransmitter release at excitatory and inhibitory synapses

Spontaneous (TTX-insensitive) neurotransmitter release in excitatory and inhibitory synapses was separately evaluated by recording miniature post-synaptic currents in the presence of bicuculline (30 µM) or CNQX (10 µM), respectively, in the bath solution. In both cases, KO neurons behaved closely similar to WT neurons regarding frequency, amplitude and kinetics of miniature post-synaptic currents (Table 1). These data suggest that Kidins220 ablation does not affect spontaneous presynaptic activity or the number and properties of postsynaptic neurotransmitter receptors.

### Evoked excitatory and inhibitory post-synaptic currents in autaptic Kidins220^−/−^ neurons have normal amplitudes and kinetic properties

In order to compare basal evoked synaptic transmission, we recorded post-synaptic currents in autaptic glutamatergic (Figures 2A) and GABAergic neurons (Figure 2F) from WT and KO embryos. As illustrated in Figure 2B and 2G, the mean amplitudes for both eEPSCs and eIPSCs were comparable, as well as the sizes of the patched neurons evaluated from the membrane capacitance. Autaptic glutamatergic neurons had capacitance values of 48.4±2.4 pF (n = 51; WT) and 52.1±2.8 pF (n = 43; KO; p>0.05, unpaired Student's t-test), while autaptic GABAergic neurons had values of 29.7±2.8 pF (n = 33; WT) and 28.9±1.8 pF (n = 28; KO; p>0.05, unpaired Student's t-test).

According to Arevalo *et al.* [13], reduced Kidins220 expression lead to increased incorporation of GluA1 subunits at the plasma membrane, which altered the AMPA receptor subunit composition at the expense of GluA2-containing complexes, ultimately enhancing AMPA receptor-mediated post-synaptic responses at Schaffer collateral – CA1 synapses. We investigated possible changes in the AMPA receptor subunit composition of autaptic KO neurons by recording eEPSCs in the presence and absence of Naspm, a blocker of GluA2-lacking AMPA receptors [21]. Local perfusion of WT autaptic neurons with Naspm (100 µM) had only a minor effect on the eEPSC amplitude (0.93±0.04 of control; n = 5), consistent with the fact that most functional AMPA receptors contain GluA2 subunits under basal conditions [13], [22]. Similarly, the eEPSC amplitude of KO neurons decreased to about the same extent upon Naspm application (0.96±0.04 of control; n = 4; p>0.05, unpaired Student's t-test), suggesting that the subunit composition of AMPA receptors was not altered in the absence of Kidins220.

This conclusion was further supported by the analysis of the kinetic properties of autaptic eEPSCs (Figure 2C–E), as the presence of GluA2 subunits was reported to slow down the decay of AMPA receptor-mediated EPSCs [23]. Consistently, we found that the delay, rise time and time constants of deactivation of both mEPSCs (Table 1) and eEPSCs (Figure 2C–E) were not altered between WT and KO neurons. Similarly, the kinetic properties of both mIPSCs (Table 1) and eIPSCs (Figure 2H–J) were not affected. Basal evoked synaptic transmission seems therefore not affected by Kidins220 ablation.

The amplitude of post-synaptic currents is determined by the linear combination of a series of parameters, such as the number of releasable synaptic vesicles, the probability of release, the vesicular neurotransmitter content and the post-synaptic receptor density. Even though evoked currents have shown equal amplitudes in WT and KO neurons, they may nevertheless hide changes in the basic parameters compensating each other. The analysis of miniature post-synaptic currents (Table 1) already confirmed that the vesicular neurotransmitter content and the number of post-synaptic receptors were unchanged in KO neurons. Cumulative amplitude profile analysis was used to estimate the vesicle release probability (Pr) and the size of the readily releasable pool (RRP) of synaptic vesicles from the current responses to high-frequency stimulation [19]. The excitatory and inhibitory responses evoked by a stimulation train of 1 s @40 Hz (Figure 3A) were plotted as cumulative amplitude profiles in Figures 3B_1_ and B_2_, respectively, and the slower linear increase at later stages of train stimulation was back-extrapolated to time zero to yield a rough estimation of the size of the RRP of synchronous release (RRP_syn_) [19]. Given the almost identical cumulative amplitude profiles for the two genotypes, RRP_syn_ and Pr were not significantly different between WT and KO for both excitatory and inhibitory neurons (Figure 3C_1_, C_2_).

### Evoked IPSCs of Kidins220^−/−^ neurons show reduced paired-pulse depression at long inter-pulse intervals

**Figure 4:**
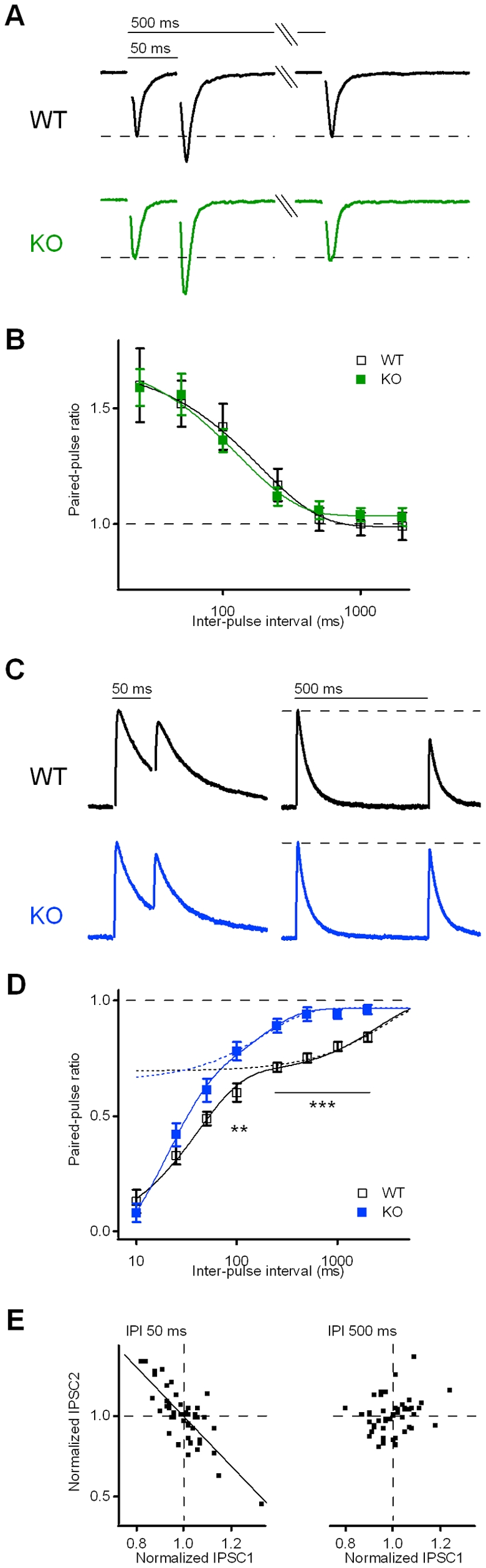
Neurons from Kidins220^−/−^ mice showed normal EPSC paired-pulse facilitation, but reduced IPSC paired-pulse depression at long inter-pulse intervals. **A**) Representative EPSC recordings in WT and KO autaptic neurons in response to paired stimuli separated by the indicated inter-pulse interval. Holding potential −86 mV. **B**) In EPSC recordings, paired-pulse protocols were applied at a stimulation frequency of 0.1 Hz, with inter-pulse intervals ranging from 25 to 2,000 ms. The paired-pulse ratio was calculated as the ratio between the second and the first amplitude, n = 11–14 for WT (open symbols) and n = 10–14 for KO (filled symbols). There was no significant difference between WT and KO cells (p>0.05; unpaired Student's t-test). Continuous lines represent best fits with a mono-exponential function. **C**) Representative IPSC recordings in WT and KO neurons in response to paired stimuli separated by the indicated inter-pulse interval. Holding potential −66 mV. **D**) In IPSC recordings, paired-pulse protocols were applied at a stimulation frequency of 0.1 Hz, with inter-pulse intervals ranging from 10 to 2,000 ms, n = 13–25 for WT (open symbols) and n = 14–22 for KO (filled symbols). Continuous lines represent best fits with a bi-exponential function, dotted lines indicate the slow component of the fit. **E**) The slow component of PPD in WT neurons appears to be independent of previous release. IPSC2 is plotted against IPSC1 for individual trials of paired-pulse stimulation. For each cell (n = 7; WT), individual IPSC amplitudes were normalized to the mean value of the recorded ensemble. The data set at IPI = 50 ms (left panel; mean PPR = 0.49) revealed an inverse relationship between IPSC amplitudes (r = −0.80; p<0.001; continuous line represents linear regression), while the data set at IPI = 500 ms (right panel; mean PPR = 0.72) for the same cells showed no such correlation (r = 0.25; p>0.05).

Since basal transmission of both excitatory and inhibitory synapses was unchanged in KO neurons, we focused our attention on various paradigms of short-term plasticity. We first applied a series of paired-pulse stimulations with inter-pulse intervals (IPI) ranging from 10 to 2,000 ms. Among the multiple mechanisms known to contribute to changes in the paired-pulse ratio (PPR = I2/I1) at short IPIs, two processes are of major importance [1]: i) residual calcium from the first pulse causes an increase in Pr of a second pulse applied at short intervals; ii) synaptic vesicle depletion generated by the first pulse leads to a decrease in the number of readily releasable vesicles for the second pulse. WT and KO excitatory neurons showed pronounced paired-pulse facilitation (PPF) at short inter-pulse intervals, i.e. the amplitude of the second EPSC response was larger than that of the first (PPR>1; Figure 4A, B). These neurons displayed low basal release probability (Pr≈0.14; Figure 3C_1_), which limits the impact of vesicle depletion, thus favoring the effect of residual calcium. PPF gradually decreased and eventually disappeared at intervals above 500 ms. Modification of the basal release probability by elevated calcium concentration (5 mM) in the bath solution caused paired-pulse depression (PPD; PPR<1) of EPSCs at all IPIs. However, also under these high-release-probability conditions, no significant differences between WT and KO cells were observed (data not shown).

On the contrary, IPSC responses were characterized by PPD in standard bath solution (containing 2 mM calcium), which is mainly due to a higher basal Pr in inhibitory synapses (Pr≈0.40; Figure 3C_2_), thus favoring vesicle depletion over residual calcium. KO responses were similar to WT at short inter-pulse intervals, but displayed a significantly smaller PPD at intervals between 100 ms and 2,000 ms (Figure 4C, D). Notably, there was essentially no PPD in KO cells at intervals above 500 ms, with the PPR approaching unity. A more detailed analysis revealed that the recovery from PPD exhibited two distinct kinetic components, namely a fast component that was similar (τ_fast_≈35 and 20 ms for WT and KO, respectively), and a slow component that was more than one order of magnitude faster for KO responses than for WT ones (τ_slow_≈2,700 and 150 ms for WT and KO, respectively). A time constant of about 2 s has been previously reported for PPD_slow_ of IPSC responses in hippocampal basket cell – granule cell synapses [24] and in collicular neurons [25]. Both studies favored a presynaptic origin of this effect and found that PPD_slow_ was independent of both extracellular calcium concentration and previous release. In fact, if PPD were caused by synaptic vesicle depletion, one would expect an inverse relationship between the first and the second amplitude during paired stimulation [26]. In other words, if the first IPSC is larger than average, the second IPSC would be smaller than average. Within the WT data set at IPI = 50 ms, some cells indeed showed the expected relationship (Figure 4E, left panel), while in other cells such relationship was not apparent. This is likely due to a low mean Pr producing less SV depletion and larger PPF. In cells showing an inverse relationship at IPI = 50 ms, no such correlation emerged at IPI = 500 ms (Figure 4E, right panel), suggesting that the pronounced PPD_slow_ in WT cells was independent of previous release.

A recent study suggested that PPD_slow_ of IPSCs recorded from rat CA1 pyramidal neurons may be mediated by the activation of GABA_C_ receptors, since it could be reversibly blocked by the application of the GABA_C_ receptor antagonist (1,2,5,6-tetrahydropyridin-4-yl)methylphosphinic acid (TPMPA) [27]. Under our experimental conditions, however, bath application of TPMPA (10–100 µM) did not lead to significant changes of the PPR of eIPSCs in WT neurons (data not shown).

In summary, these data show that the extent of PPD_slow_ in KO neurons was dramatically reduced, suggesting that Kidins220 is required for this form of synaptic plasticity.

### Evoked EPSCs of Kidins220^−/−^ neurons show normal post-tetanic potentiation and synaptic depression

**Figure 5:**
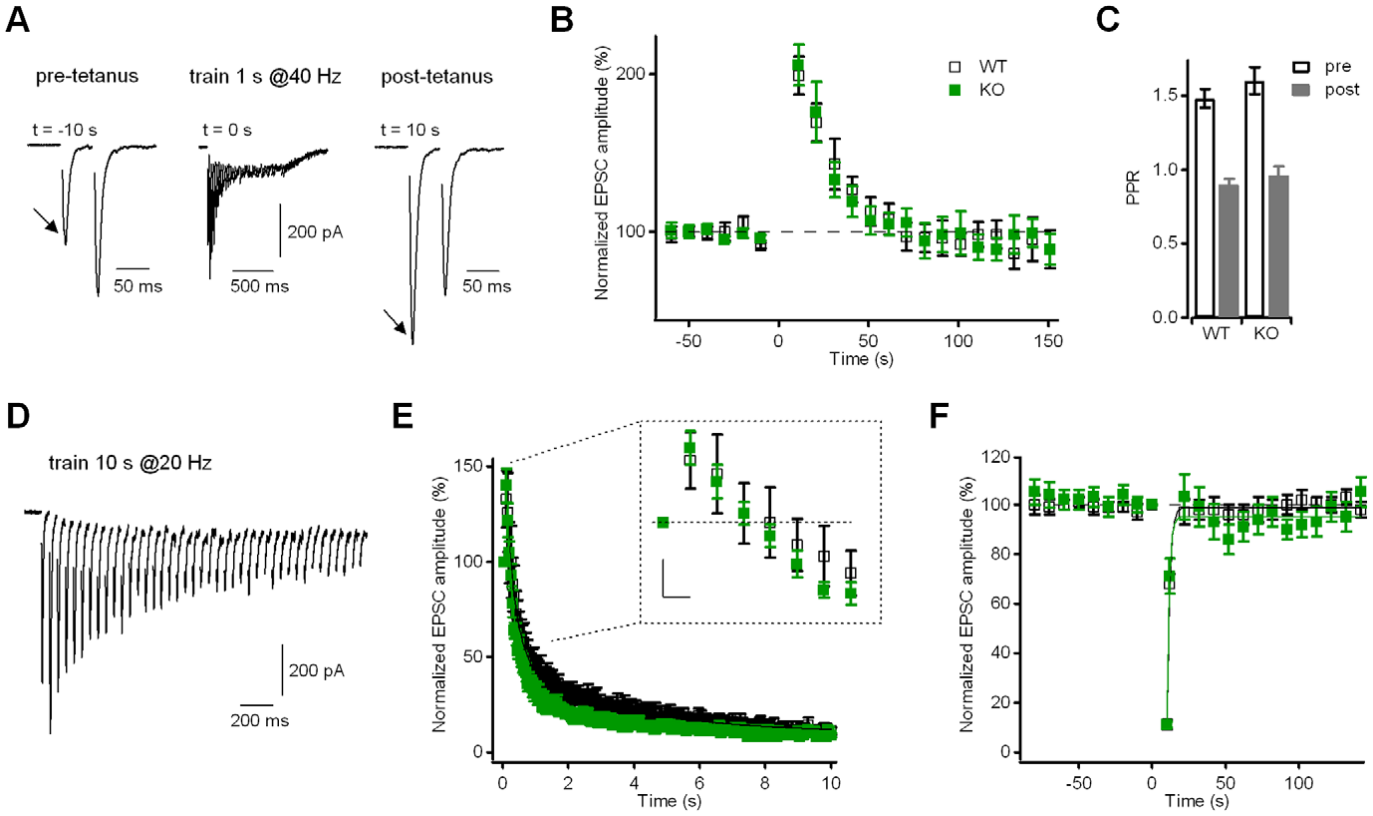
Neurons from Kidins220^−/−^ mice showed normal post-tetanic potentiation and synaptic depression of evoked EPSCs. **A**) EPSCs recorded using a paired-pulse protocol (IPI = 50 ms) are presented before (left trace) and after (right trace) the application of a 1-s stimulation train at 40 Hz (middle trace). The increase of the EPSC1 amplitude (arrows) is connected to a decrease of the paired-pulse ratio. Holding potential −86 mV. **B**) Time course of post-synaptic currents (in % of baseline) recorded at a stimulation frequency of 0.1 Hz. Tetanic stimulation (as in **A**) was applied at t = 0 s. EPSCs displayed post-tetanic potentiation with a peak at 10 s after the end of tetanic stimulation. There was no significant difference between WT and KO cells (n = 22 for both groups; p>0.05, unpaired Student's t-test). **C**) The paired-pulse ratio of EPSC recordings for both WT and KO neurons (n = 22 for both groups; p>0.05, unpaired Student's t-test) changes from facilitation in the baseline condition (pre) to depression at the 10-s time point after tetanic stimulation (post). **D**) Representative EPSC trace in response to a 10-s stimulation train at 20 Hz to induce synaptic depression. Only the first 2 s corresponding to 40 pulses are shown for clarity. Holding potential −86 mV. **E**) Time course of EPSC responses during the application of a 10 s @20 Hz train. Data were normalized to the amplitude of the first current response in the train. There was no significant difference between WT and KO cells (n = 16 for both groups; p>0.05, unpaired Student's t-test). Continuous lines represent best fits with a bi-exponential function. The inset illustrates the transient increase of the EPSC amplitude during the first pulses of the train on an expanded scale. Scale bars 50 ms/20%. **F**) Recovery from depression of EPSC responses was followed at a stimulation frequency of 0.1 Hz. All data points were normalized to the amplitude of the first current response in the train (applied at time point 0). There was no significant difference between WT and KO cells (p>0.05, unpaired Student's t-test). Lines represent best-fits with a mono-exponential function.

Brief tetanic stimulation induces a second type of short-term plasticity involving a temporary accumulation of cytosolic calcium within the presynaptic terminal. In excitatory neurons, a train of 40 stimuli in 1 s caused post-tetanic potentiation (PTP) of the eEPSC amplitude (Figure 5A, B). PTP reached its maximum of about 100% current increase at 10 s after the stimulus train and then decayed with a time constant of 26 s. Postsynaptic current potentiation was accompanied by a strong reduction of the PPR of paired recordings: PPR changed from facilitation in the pre-tetanus period to depression at the maximum of PTP (Figure 5C), indicating that the current increase was due to a Pr increase. Responses of KO neurons were similar to WT regarding PTP amplitude, decay kinetics, PPR change and in the time course of EPSCs during the train (see also the cumulative amplitude profile in Figure 3B_1_). The same tetanic stimulation (1 s @40 Hz) applied to inhibitory neurons did not lead to a subsequent variation of the eIPSC amplitude. Similarly to what has been observed in excitatory cells, KO behaved identically to WT in terms of responses both during (Figure 3B_2_) and after the stimulation train (data not shown).

Synaptic depression in glutamatergic synapses was evaluated from the EPSC responses to a 10 s @20 Hz train (Figure 5D, E), and subsequent recovery from depression was followed at a stimulation frequency of 0.1 Hz (Figure 5F). Excitatory neurons of both genotypes showed a transient increase of the EPSC amplitude during the first pulses of the train, followed by a progressive decay to a quasi-stationary level (Figure 5E and inset therein). Double-exponential fitting of the data gave similar values for the time constant of fast decay τ_fast_ (221±22 ms for WT; 195±25 ms for KO; p>0.05, unpaired Student's t-test), for the time constant of slow decay τ_slow_ (3.3±0.4 s for WT; 2.3±0.4 s for KO; p>0.05, unpaired Student's t-test) and for the steady-state EPSC I_ss_ (9.4±0.8% for WT; 8.4±0.7% for KO; p>0.05, unpaired Student's t-test). Recovery from depression (Figure 5F) was already complete at the 12-s time point after the train and could be well described by a single exponential function with similar time constants of 1.9±0.1 s (WT) and 1.6±0.3 s (KO).

### Evoked IPSCs of Kidins220^−/−^ neurons show faster recovery from synaptic depression

**Figure 6:**
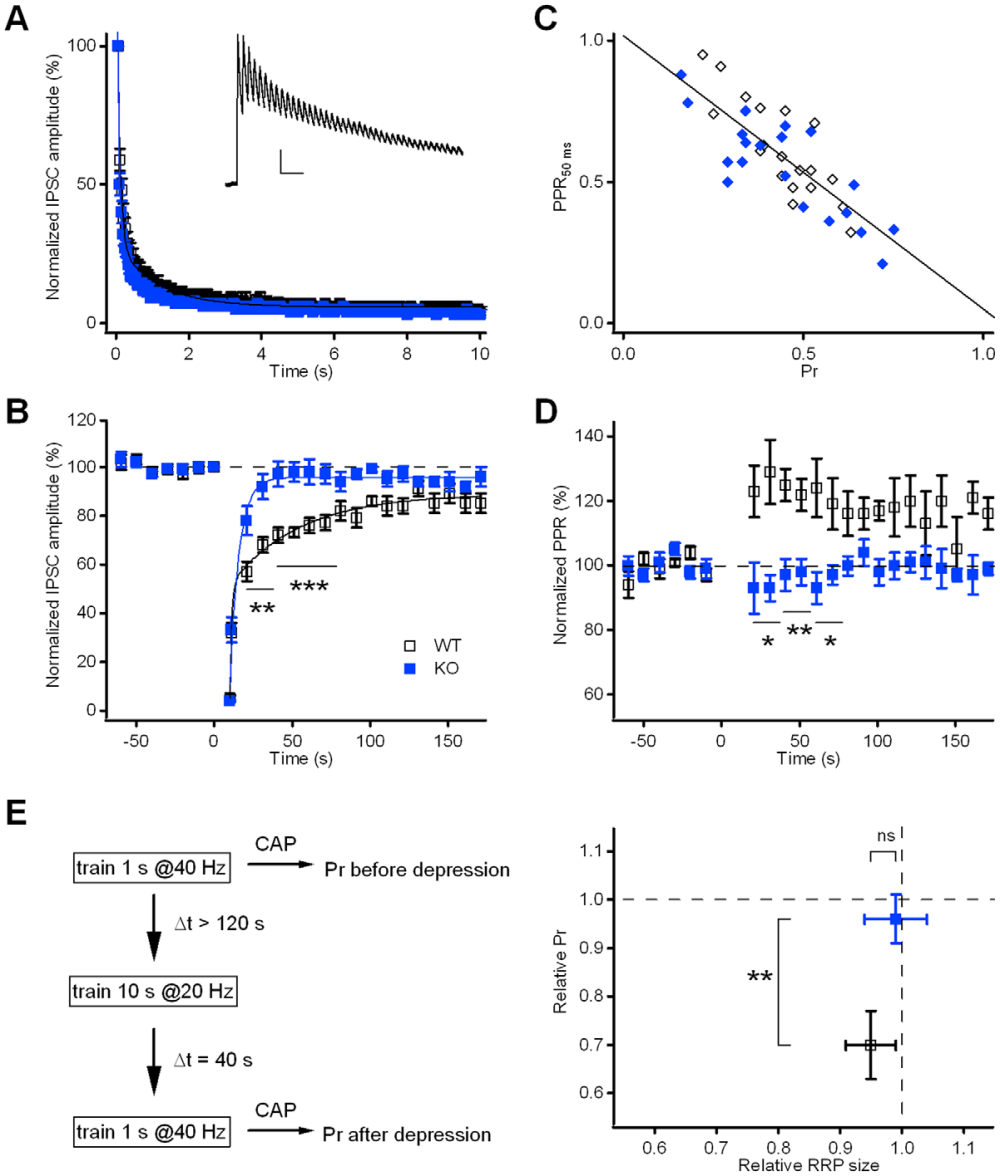
Neurons from Kidins220^−/−^ mice showed normal train-induced depression of IPSCs, but faster recovery from depression. **A**) Time course of IPSC responses during the application of a 10-s stimulation train at 20 Hz to induce synaptic depression. Data were normalized to the amplitude of the first current response in the train, n = 15 for WT and n = 11 for KO. There was no significant difference between WT and KO cells (p>0.05; unpaired Student's t-test). Lines represent best-fits with a bi-exponential function. The inset shows a representative current trace (only the first 2 s corresponding to 40 pulses are shown for clarity). Scale bars 100 pA/200 ms. Holding potential −66 mV. **B**) Recovery from depression of IPSC responses was followed at a stimulation frequency of 0.1 Hz. All data points were normalized to the amplitude of the first current response in the train (applied at time point 0). Lines represent best-fits with a bi-exponential function. **C**) Inverse relationship between the paired-pulse ratio (IPI = 50 ms) of baseline IPSC recordings immediately before tetanic stimulation and the vesicle release probability (Pr), obtained from cumulative amplitude profile analyses (see Figure 3). Pooled data points from 20 WT cells (open diamonds) and 20 KO cells (closed diamonds) were fitted with a linear function (continuous line; slope −0.97; r = −0.80; p<0.001). **D**) Time course of the paired-pulse ratio (PPR; IPI = 50 ms) of IPSC recordings. The PPR at every time point was normalized to the mean PPR before the stimulation train (applied at time point 0), n = 9 for WT and n = 8 for KO. **E**) Experimental scheme (left panel) illustrating the application of 1-s stimulation trains at 40 Hz before synaptic depression and during recovery from depression to estimate Pr using cumulative amplitude profile (CAP) analysis. Relative changes of Pr and RRP_syn_ for 8 WT cells (baseline Pr 0.45) and 4 KO cells (baseline Pr 0.47) are shown in the right panel.

Similarly to excitatory synapses, also for GABAergic responses, the decay of IPSC amplitude during the application of a stimulation train lasting 10 s @20 Hz was not significantly different between WT and KO neurons (Figure 6A; τ_fast_, 92±26 ms and 76±21 ms; τ_slow_, 1.7±0.4 s and 1.2±0.3 s; I_ss_, 4.8±0.7% and 4.2±0.6%, respectively; p>0.05, unpaired Student's t-test). Interestingly, a phenotypic difference became again evident in the recovery from synaptic depression (Figure 6B), similarly to the results of the paired-pulse recordings of inhibitory responses (Figure 4D). The time course of recovery in WT cells was slow and exhibited two kinetically distinct components with time constants of 1.2±0.4 s (amplitude contribution 56%) and 44.9±13.1 s (amplitude contribution 44%), respectively, which are in close agreement with the values reported for hippocampal basket cell – granule cell synapses [24]. In contrast, KO responses recovered to their pre-stimulation level much earlier (already 30 s after the end of the train), with a fast time constant of 0.4±0.3 s (amplitude contribution 22%) and a slow time constant of 7.7±5.7 s (amplitude contribution 78%). Thus, similarly to PPD, although on a different time-scale, eIPSCs in KO cells recovered faster from train-induced depression than in WT cells, apparently due to a dramatic (almost 6-fold) reduction in the time constant of the slow component of recovery. We investigated the possibility that the slow component was due to a temporary reduction of Pr, by comparing the PPR of IPSC responses before depression and during recovery. According to the depletion model of depression, the extent of PPD at short inter-pulse intervals is dependent on the initial probability of release [1]. Indeed, the data in Figure 6C reveal an inverse relationship between the mean PPR_50ms_ under baseline conditions and the Pr value obtained from the cumulative amplitude profile analysis (Figure 3B_2_). With the slope of the line fit close to −1, variations of PPR could be easily related to opposite changes of the probability of release. During recovery from depression, the PPR of KO responses returned immediately to the value before the train (Figure 6D); instead, the PPR of WT currents increased by 25% for about 60 s after the train and then slowly, although with high variability, approached the baseline level. Interestingly, the time-course of PPR was the mirror-image of the depression of eIPSC amplitudes during the slow phase of recovery in Figure 6B. If one assumes that a 25% PPR increase corresponds to an approximate 25% decrease of Pr (Figure 6C), the slow component of synaptic depression in WT cells can be fully accounted for by a temporary reduction of Pr after the train.

Moreover, we used a second independent approach to get directly hands on possible changes of Pr during recovery from depression. Cumulative amplitude profile analysis on IPSC responses to tetanic stimulation was performed before and after the induction of synaptic depression, as illustrated in Figure 6E (left panel). Pr was determined twice on the same cell: first under baseline conditions and a second time 40 s after the end of the depression train, i.e. at the time point of the maximal difference between WT and KO amplitudes during recovery from depression (see Figure 6B). The summary of the Pr ratios (Figure 6E, right panel) illustrates that Pr in KO cells remained invariant, while WT cells had, on average, 30% lower Pr values after depression, in the absence of significant changes in RRP size ratios. These data fully confirm the results obtained from the PPR analysis and further support the conclusion that the slow component of recovery from synaptic depression in WT cells is caused by a temporary reduction of Pr after the train. The fast recovery of eIPSC amplitude and PPR/Pr in KO neurons suggests that Kidins220 favors the expression of this type of short-term plasticity in WT cells.

## Discussion

In this study we present a comprehensive description of synaptic transmission and plasticity in cultured hippocampal neurons isolated from embryonic Kidins220^−/−^ mice, with the aim to assess the functional consequences of the chronic ablation of this scaffold protein. Our studies were conducted in the well-established autaptic culture system, in addition to low-density neuronal networks, which allowed a precise quantitative evaluation of synaptic parameters. KO neurons did not show any changes in either AMPA or GABA receptor-mediated basal synaptic transmission. However, our data revealed a novel role of Kidins220 in GABAergic short-term synaptic plasticity.

### Basal excitatory neurotransmission and short-term plasticity are not affected in Kidins220^−/−^ neurons

We evaluated basal glutamatergic synaptic transmission in KO neurons by two independent types of recordings: i) miniature EPSCs in low-density neuronal networks and ii) evoked EPSCs triggered by brief depolarization of autaptic neurons. In both cases, there was no significant difference in EPSC amplitudes between WT and KO cells. Previous work showed that reduced Kidins220 expression, either chronically in Kidins220^+/−^ mice or acutely by RNA silencing in primary neuronal cultures, lead to increased basal excitatory transmission [13], [15]. Arevalo *et al.* [13] proposed that Kidins220 depletion increases EPSCs and alters the subunit composition of synaptic AMPA receptors by favoring the selective incorporation of new GluA1 subunits into the plasma membrane. Our data do not confirm these observations, since EPSC amplitudes, decay kinetics and sensitivity towards a GluA2-lacking AMPA receptor blocker were unaltered in KO neurons. These divergent results may be due to differences in the experimental systems used for EPSC recordings, namely the complete and constitutive absence of Kidins220 in KO neurons *versus* reduced Kidins220 expression in Kidins220^+/−^ mice or shRNA-treated neuronal cultures and the use of dissociated hippocampal neurons in autaptic or low-density culture *versus* acute or organotypic hippocampal slices.

We also analyzed the responses to three different types of short-term plasticity, but we did not find significant differences in paired-pulse facilitation, post-tetanic potentiation and train-induced synaptic depression between WT and KO neurons. These results suggested that activity-dependent alterations in calcium homeostasis and vesicle dynamics were not affected by the lack of Kidins220 in excitatory neurons. Constitutive Kidins220 ablation therefore does not appear to affect basal excitatory neurotransmission and short-term plasticity in hippocampal neurons.

### Short-term plasticity of inhibitory neurotransmission is altered in Kidins220^−/−^ neurons

A recent report pointed to a role of Kidins220 in the regulation of inhibitory neurotransmission in rat hippocampal neurons [7]. Increased Kidins220 expression lead to higher amplitude and higher frequency of mIPSCs recorded from pyramidal excitatory neurons, while the opposite effect was observed with decreased expression. The fact that the manipulation of Kidins220 expression affected mIPSC frequency and the intensity of GAD65 puncta suggested a predominant presynaptic mechanism. In support of this hypothesis, these alterations were not associated with changes in the number or subunit composition of postsynaptic GABA_A_ receptors. In contrast, our measurements of mIPSCs and autaptic eIPSCs suggested that basal GABAergic synaptic transmission was unchanged in KO neurons. Indeed, neither amplitude nor frequency of mIPSCs were affected by Kidins220 ablation. Again, these divergent results may be caused by differences in the experimental conditions. In fact, experiments in Sutachan *et al.* [7] were performed at 11–12 div on rat hippocampal neurons after a 10-d period of reduced Kidins220 expression (protein level 30% of control). Our mIPSC measurements were done at 14–17 div on mouse hippocampal neurons completely lacking Kidins220. In addition to spontaneous GABA release, we also investigated for the first time the role of Kidins220 in evoked inhibitory transmission. Our analyses of autaptic eIPSCs did not reveal any differences between WT and KO neurons regarding amplitude, kinetics, RRP size and Pr, supporting the view that the synaptic parameters contributing to basal GABAergic neurotransmission are unaffected by Kidins220 ablation.

Importantly, our study unveiled specific differences in the response to paired-pulse stimulation and in the recovery from train-induced depression, in line with a novel role of Kidins220 in GABAergic short-term plasticity. PPD of eIPSC responses exhibited two kinetically distinct components, which presumably relied on different mechanisms. The fast component was normal in KO neurons, while the slow component was significantly accelerated, leading to reduced PPD at long inter-pulse intervals. Previously, PPD_slow_ with similar time constants was described in GABAergic synapses between basket cells and granule cells in the rat dentate gyrus [24], in mouse cortical cultures [28] and in rat collicular cultures [25]. All these studies attributed this form of plasticity to a release-independent inhibition of exocytosis, but the underlying mechanism is still unknown (see ref. [24] for a detailed discussion of candidate mechanisms). In agreement with other reports [24], [29], presynaptic receptor activation was not involved in PPD_slow_ in WT cells, since all recordings were performed in the presence of the GABA_B_ receptor antagonist CGP 55845, and the GABA_C_ receptor blocker TPMPA had no effect on PPR (but see ref. [27]). Furthermore, PPD_slow_ is unlikely to be caused by depletion of releasable vesicles after the first pulse, since the extent of PPD was independent of previous release at long, but not at short IPIs (Figure 4E), and insensitive to manipulations of the average Pr [24], [25]. Possible changes in presynaptic GABA release can be detected by comparing the coefficients of variation (CV) of the first and the second IPSC during PPD_slow_ [24], [29]. However, the CV of IPSC amplitudes under baseline conditions and the Pr obtained from the analysis of cumulative amplitude profiles were not sufficiently correlated in our data sets (data not shown). Consequently, the CV analysis of paired-pulse recordings did not provide conclusive evidence for a reduction of presynaptic GABA release in PPD_slow_.

It is important to note the common ground between the recovery from PPD and train-induced depression: both exhibited bi-exponential kinetics in WT cells, although on different time scales, and in both cases, the fast component was normal in KO cells, but the slow component was strongly reduced. In the recovery from depression induced by a stimulus train of 10 s at 20 Hz, the slow component in WT cells can be fully explained by a transient reduction of Pr, which appears to be absent in KO cells. The processes underlying vesicle release and replenishment upon prolonged stimulation at high frequency do not seem to be affected by Kidins220 ablation. The refilling of the RRP after the train is likely reflected by the fast component of recovery, which was normal in KO neurons. Likewise, the dual time course of synaptic depression during train stimulation was also very similar between WT and KO cells. Here, the fast component may be caused by an accumulation of the events underlying PPD_fast_ (at IPI = 50 ms), while the slow phase likely reflects a mechanism counteracting RRP depletion by vesicle recruitment from a recycling pool [24], [28]. The preservation of normal vesicle release and replenishment in response to prolonged stimulation further supports the idea that the absence of Kidins220 may selectively affect the transient reduction of Pr. Considering that vesicle depletion could be excluded for the slow components of recovery from both train-induced and paired-pulse depression, one may speculate that Kidins220 may be involved in a common mechanism determining an activity-dependent, transient reduction of GABA release.

How may such reduction of release probability be achieved? Wu and Borst [30] described a similar phenomenon in the excitatory calyx of Held synapses, in which recently recruited vesicles showed a transient Pr decrease. This was caused in part by the inactivation of calcium currents, but mostly by mechanisms downstream of calcium entry, e.g. the larger distance between docked vesicles and voltage-gated calcium channels or the lower calcium sensitivity of the recruited vesicles [30]. Thus, Kidins220 could slow down the recovery of a normal Pr level for synaptic vesicles recently recruited to the RRP. Although Kidins220 has never been localized specifically at release sites, its interaction with septin5 [31] may indicate its involvement in the regulation of vesicle release. Septin5 can bind to ternary SNARE complexes through its direct association with syntaxin and inhibits exocytosis in secretory cells [32], [33]. In the calyx of Held synapses, septin5 is involved in the regulation of the coupling of calcium influx to neurotransmitter release [34]. Septin-containing filaments surround synaptic vesicles in the nerve terminal and appear to have an inhibitory role in the positional priming at the pre-fusion stage. Since Park *et al.* [31] suggested that septin5 may interact with syntaxin and Kidins220 simultaneously, we propose a model whereby Kidins220 may be involved in these regulatory processes. In the absence of Kidins220, the molecular “brake” may be partly released allowing the fast priming and release of newly recruited vesicles.

### Conclusions

In contrast to results obtained using neurons with altered Kidins220 expression, the constitutive absence of Kidins220 did not affect basal synaptic transmission either in excitatory or inhibitory synapses. However, distinct phenotypic alterations were specifically detected in inhibitory short-term synaptic plasticity, despite the fact that Kidins220 is expressed in comparable amounts in both glutamatergic and GABAergic neurons [7] (this study). These findings suggest that neurons may modulate the strength of basal synaptic transmission by altering Kidins220 protein levels, but they are able to compensate for its constitutive absence possibly by means of their intrinsic plastic properties. Instead, Kidins220 appears to be required for the activity-dependent transient decrease of release probability in inhibitory neurons. Our previous study provided evidence for an involvement of the protein in the neurotrophin signaling pathway at glutamatergic synapses, as the potentiation of EPSCs induced by acute BDNF treatment was impaired in Kidins220^−/−^ excitatory neurons [6]. Thus, it appears that Kidins220 fulfills distinct functions in the two types of synapses. Since Kidins220 coordinates diverse regulatory functions via multiple protein-protein interactions, it is expected that such differences are related to the identity of the specific interaction partners, which may differ between glutamatergic and GABAergic synapses, and in different physiological situations.

Which could be the physiological role of Kidins220 in terms of the electrical activity of neuronal circuits? Considering that Kidins220 limits the efficacy of SV recovery in inhibitory synapses and increases the intensity and duration of synaptic depression, it is possible to predict that the overall firing rate and the synchronized activity of the neuronal network will be decreased by its deletion. The depression rate, frequency dependence and recovery kinetics are fundamental features of inhibitory neurons and, at the network level, temporally and spatially shape the areas of excitation. Our data suggest a role of Kidins220 in prolonging the temporal windows for depression in inhibitory synapses, potentially enhancing the high band-pass filtering properties of neuronal circuits. Thus, under physiological conditions, Kidins220 could make the information transfer more reliable by enhancing the synchronized activity and reducing the signaling noise due to random spiking activity.

Moreover, GABAergic dysfunctions such as those displayed by KO neurons could potentially shift the balance of excitatory and inhibitory transmission in a neuronal network. An imbalance between excitation and inhibition is at the basis of several neuropathological conditions such as epilepsy or autism spectrum disorders. The embryonic lethality of the Kidins220^−/−^ strain has prevented the study of inhibitory circuits in adult mutant animals. The generation of mouse lines bearing a nervous system-specific deletion of Kidins220 will therefore be instrumental to define how Kidins220 ablation might impact on cortico-hippocampal excitability and plasticity.

## Methods

### Ethics statement

All experiments were performed in accordance with the European Community Council Directive dated November 24, 1986 (86/609/EEC) and approved by the Italian Ministry of Health.

### Generation of Kidins220^−/−^ mice and primary hippocampal cultures

The generation of the Kidins220^−/−^ strain was described in Cesca *et al.* [6]. All embryos used in this study were obtained from crosses of Kidins220^+/−^ mice on the C57BL/6 background. Mice were mated overnight and separated in the morning. The development of the embryos was timed from the detection of a vaginal plug, which was considered day 0.5.

Hippocampi were dissected from wild type and Kidins220^−/−^ E18.5 embryo littermates obtained from crossing Kidins220^+/−^ mice. Briefly, hippocampi were dissected in ice-cold PBS, incubated with trypsin (0.125%) for 15 min at 37°C, and mechanically dissociated. Neurons were then resuspended and plated on poly-D-lysine/laminin coated glass coverslips, in Neurobasal medium containing 10% horse serum, 2 mM glutamine and antibiotics (plating medium). After 3 hours, the medium was removed and replaced with Neurobasal containing 2% B27 supplement, 2 mM glutamine and antibiotics (maintenance medium). Autaptic cultures were prepared as previously described [18]. Briefly, dissociated neurons were plated at very low density (20 cells mm^−2^) on microdots (40–300 µm diameter) obtained by spraying poly-D-lysine (0.14 mg ml^−1^) on dishes pre-treated with 0.15% agarose.

### Immunocytochemistry

Cells were fixed with 4% PFA/20% sucrose in PBS for 15 min at RT and permeabilized with 0.1% Triton ×100 in PBS for 5 min at RT. Samples were blocked for 30 min in IF buffer (2% BSA, 10% goat serum in PBS). Primary and secondary antibodies were diluted in IF buffer and incubated for 45 min at RT. The following primary antibodies were used: monoclonal anti-Kidins220 [11], polyclonal anti-vesicular GABA transporter (vGAT, AB5062P, Millipore), polyclonal anti-vesicular Glutamate transporter 1 (vGlut1, AB5905 Millipore). Fluorescently-conjugated secondary antibodies for immunofluorescence were from Molecular Probes (Invitrogen).

Images were acquired at an inverted Leica TCS SP5 AOBS TANDEM confocal microscope equipped with a 40×/1.25-0.75 HCX PL APO Oil objective. Images were visualised and processed by using the Leica LAS AF, ImageJ and Adobe Photoshop CS3 softwares.

### Patch-clamp recordings and data analysis

Patch-clamp experiments on cultured hippocampal neurons were conducted between 10 and 17 days *in vitro* (div). Recordings were performed in the whole-cell configuration using an EPC-10 patch clamp amplifier (HEKA Elektronik, Lambrecht, Germany). Data acquisition and analysis were done using the PatchMaster and FitMaster programs (HEKA Elektronik, Lambrecht, Germany), respectively. Data of evoked post-synaptic currents were low-pass filtered at 3 kHz and acquired at 10 kHz sample frequency, while data of miniature post-synaptic currents were filtered at 2 kHz and acquired at 5 kHz. Patch pipettes were fabricated from borosilicate glass capillaries with a Narishige PC-10 puller and had final resistances of 4.5–5.5 MΩ when filled with the standard pipette solution used for evoked post-synaptic currents. This solution contained (in mM) 126 K-gluconate, 4 NaCl, 1 MgSO_4_, 0.02 CaCl_2_, 0.1 1,2-bis(2-aminophenoxy)ethane-*N,N,N′,N′*-tetraacetic acid (BAPTA), 3 Na_2_ATP, 0.1 NaGTP, 15 glucose, 5 HEPES, pH 7.30 adjusted with KOH. The bath solution contained (in mM) 140 NaCl, 4 KCl, 2 CaCl_2_, 1 MgCl_2_, 10 glucose, 10 HEPES, pH 7.30 adjusted with NaOH. D-(−)-2-amino-5-phosphonopentanoic acid (D-AP5; 50 µM) and CGP 55845 (5 µM) were routinely added to block NMDA receptors and GABA_B_ receptors, respectively. Toxins were purchased from Tocris (Bristol, UK), unless otherwise indicated, and were supplemented as concentrated stock solutions. For recordings of miniature post-synaptic currents, the pipette solution contained an equimolar amount of KCl instead of K-gluconate, and the bath solution was supplemented with 0.3 µM tetrodotoxin (TTX). To isolate AMPA receptor-mediated or GABA_A_ receptor-mediated responses, the bath solution contained (−)-bicuculline methiodide (30 µM) or 6-cyano-7-nitroquinoxaline-2,3-dione (CNQX; 10 µM; Sigma, Milan, Italy), respectively. To test for the effect of the GluA2-lacking AMPA receptor blocker 1-naphtyl acetyl spermine (Naspm), autaptic neurons were locally perfused at a constant rate of 150 µl min^−1^, using a perfusion pipette with five inlets and a single outlet. Following Naspm application and washout, each cell was exposed to CNQX via an independent perfusion channel, which completely and reversibly abolished EPSCs (data not shown). Experiments were performed at room temperature (22–24°C).

#### Recordings of miniature and evoked post-synaptic currents

Recordings with leak currents >100 pA (at −86 mV; all potentials off-line-corrected for liquid junction potentials) or series resistance >20 MΩ were discarded. Miniature post-synaptic currents (mEPSCs and mIPSCs) were generally recorded at a holding potential of −75 mV, using neuronal cultures between 13 and 17 div. The Mini Analysis program (Synaptosoft Inc., Fort Lee (NJ), USA) was used for data analysis.

Evoked excitatory post-synaptic currents (eEPSCs) were recorded exclusively from autaptic glutamatergic cells (10–14 div), while evoked inhibitory post-synaptic current (eIPSC) responses were derived either from autaptic GABAergic cells or from extracellular stimulation of GABAergic synapses in neuronal networks (11–15 div). Subsequent analysis did not reveal significant differences (data not shown), therefore the two eIPSC data sets were pooled. Autaptic postsynaptic currents were evoked by applying a brief depolarization (0.5 ms; +24 mV) from a holding potential of −86 mV (eEPSCs) or −66 mV (eIPSCs). Mean autaptic amplitudes (Figures 2B, G) were taken from the first recording protocol (generally 2–3 minutes after establishing the whole-cell configuration). For extracellular synaptic stimulation in neuronal networks, current pulses of 0.2 ms duration and variable amplitude (10–40 µA) were delivered by an isolated pulse stimulator (A-M Systems, Carlsborg (WA), USA). The distinction between eEPSCs and eIPSCs in autaptic neurons was based on their decay kinetics, reversal potentials and, retrospectively, by the application of specific antagonists of post-synaptic receptors (see above).

The evaluation of kinetic parameters of post-synaptic currents included delay times, current activation and current deactivation. Analysis of eEPSC activation was complicated by the fact that the current onset was masked by the deactivation phase of large transient sodium currents. Therefore, the delay time was generally determined as the time between the stimulus and the peak of the post-synaptic current response. One has to consider that this value does not represent the synaptic delay (the time between stimulus and current onset), but also includes the rising phase of current activation. The rise rate (in s^−1^) as a measure of the eEPSC activation kinetics was determined from the slope of the EPSC's rising phase (in nA s^−1^) normalized to the EPSC amplitude (in nA). For eIPSCs, which were not confounded by inward sodium currents, we measured the rise time from 10% to 90% of the IPSC amplitude. Finally, the time constants of current deactivation were determined by fitting the eEPSC decay with a mono-exponential function and the eIPSC decay with a bi-exponential function.

#### Paired-pulse and train stimulation

Paired-pulse protocols consisted of two depolarization pulses separated by variable inter-pulse intervals (IPI) ranging from 10 to 2,000 ms. For each cell, the mean paired-pulse ratio at a given IPI was determined from the responses to at least 6 consecutive trials applied at 0.1 Hz. Moreover, a paired-pulse protocol (IPI 50 ms) was routinely applied in connection with train stimulation protocols. The response to the first stimulus (I1) was used to follow the current amplitudes under baseline conditions and during recovery from train-induced variations. At the same time, the response to the second stimulus (I2) allowed to calculate the paired-pulse ratio PPR = I2/I1 and infer possible changes in release probability. The coefficient of variation (CV) was calculated as the ratio between the standard deviation (sd) and the mean value of current amplitudes. Recordings of recovery from train-induced depression, in which the current amplitude did not reach at least 80% of the baseline value within 180 s, were routinely excluded from analysis. For the time-course of PPR during recovery from train-induced depression (Figure 6D), recordings displaying high variability in baseline PPR (CV>10%) were excluded from the data sets.

#### Cumulative amplitude profile analysis

The size of the readily releasable pool of synaptic vesicles during synchronous release (RRP_syn_) and the probability of SV release from the RRP (Pr) were estimated using cumulative amplitude profile analysis [19]. The cumulative amplitude profile was constructed by summing up the current amplitudes for 40 consecutive stimuli applied at 40 Hz (for both excitatory and inhibitory synapses). This analysis assumes that depression during the steady-state phase is limited by a constant recycling of SVs and equilibrium occurs between released and recycled SVs. The steady-state phase of depression during the train was determined by linear fitting of the last 16 data points (between 600 and 1,000 ms). Back-extrapolation of the line fit to time 0 yielded the size of RRP_syn_ (intercept with the y-axis), and Pr was calculated from the ratio between the amplitude of the first stimulus and RRP_syn_ (Pr = I1/RRP_syn_).

#### Statistical analyses

Data are represented as means ± standard error of the mean (for the number n of cells) throughout text and figures. Statistical comparisons were made using unpaired Student's t-test; p-values<0.05 were considered significant, with (*) indicating 0.01<p<0.05, (**) 0.001<p<0.01, (***) p<0.001.
